# Tattoo pruritus successfully treated with ruxolitinib: A case series

**DOI:** 10.1016/j.jdcr.2024.02.020

**Published:** 2024-03-05

**Authors:** Julianna Shin, Marie Leger

**Affiliations:** a Entière Dermatology, New York, New York; b Assistant Professor of Dermatology at Mount Sinai, Entière Dermatology, New York, New York

**Keywords:** contact dermatitis, red tattoo allergy, tattoo pruritus, tattoo reaction, topical JAK inhibitor

## Introduction

Tattooing, a worldwide cultural phenomenon, serves as a unique form of self-expression and personal narrative. However, tattoos occasionally intersect with dermatological challenges, notably in allergic reactions to red tattoo ink. These reactions contribute significantly to the overall rate of adverse tattoo-related incidents. Beyond their incidence, the pruritic symptoms associated with these reactions are severely distressing and profoundly affect individuals' quality of life. While established treatment options have shown promise, they can sometimes require prolonged topical steroid use, long term systemic medication, or disfiguring procedural interventions, particularly for skin of color patients.

## Case report

### Case 1

**Fig 1:**
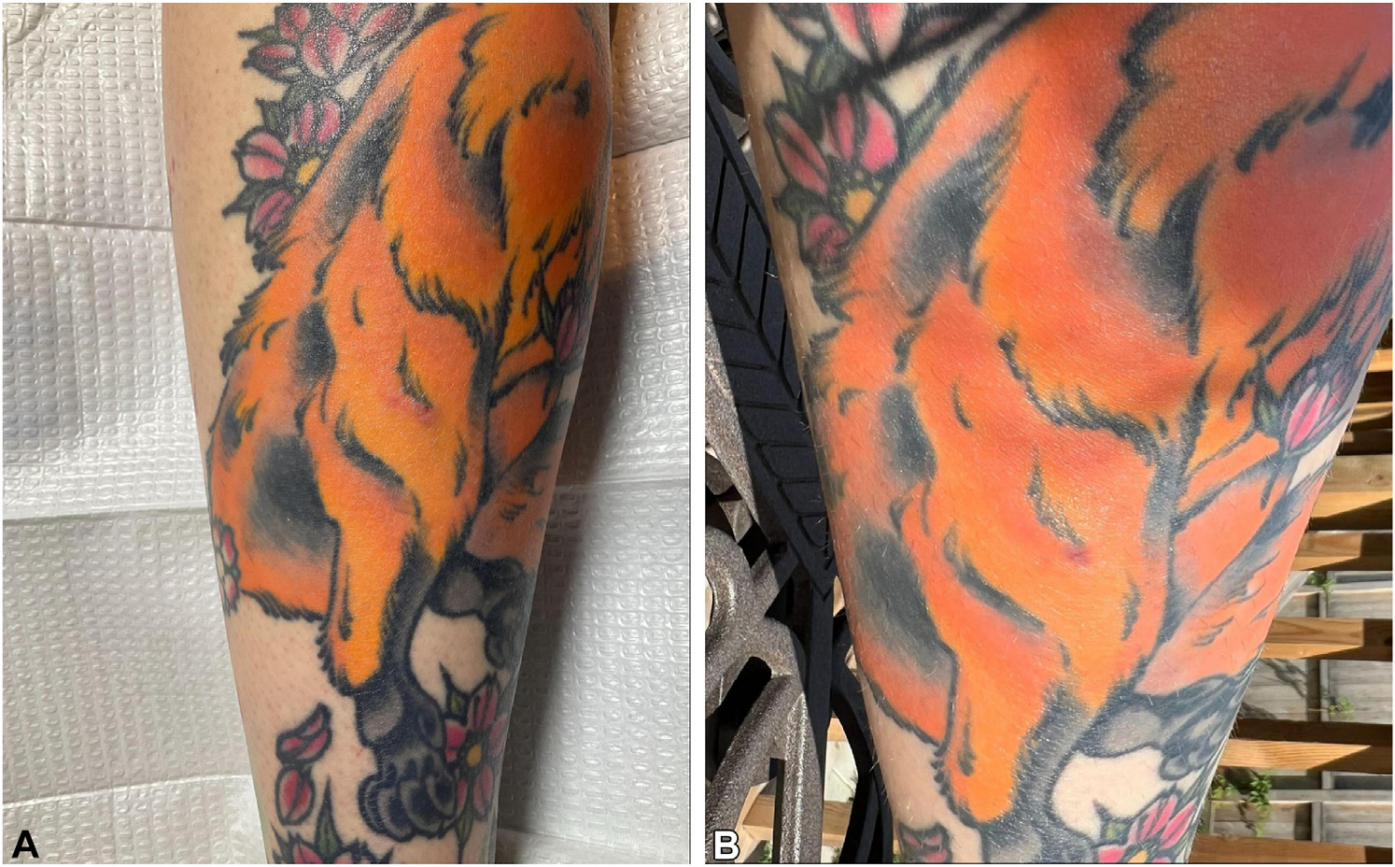
**A,** Case 1 initial presentation demonstrating 1 *red* erythematous plaque, (**B**) presentation at follow-up of biopsy region.

A 30-year-old female presented to dermatology for the evaluation of 2 raised, scaly, and severely pruritic plaques in the orange portion of a left proximal calf tattoo present for 18 months. She had obtained the tattoo 5 years prior without complications, but reported “delayed healing.” However, symptoms and reactions were absent on the other red tattoos. Furthermore, the patient denied any reactions from previous tattoos. The patient reported no significant dermatologic and medical history. A physical exam revealed erythematous nodules and eczematous plaques with hyperpigmentation distributed on the orange tattoos of the left proximal calf (Fig 1, *A*). A punch biopsy was performed which showed spongiotic dermatitis. The patient was prescribed ruxolitinib 1.5% cream to treat the symptoms. At follow-up at 6 weeks, the lesions were still raised, but the plaques were healed and the patient reported the pruritus had completely resolved (Fig 1, *B*). The patient reported that her pruritus resolved 5 days after initiating topical ruxolitinib while the raised plaques started to improve after 2 weeks. The patient reported that the raised lesions continued to resolve after she discontinued ruxolitinib after 5 weeks. The patient denied relapse of symptoms after discontinuation and reported the texture was completely healed.

### Case 2

**Fig 2:**
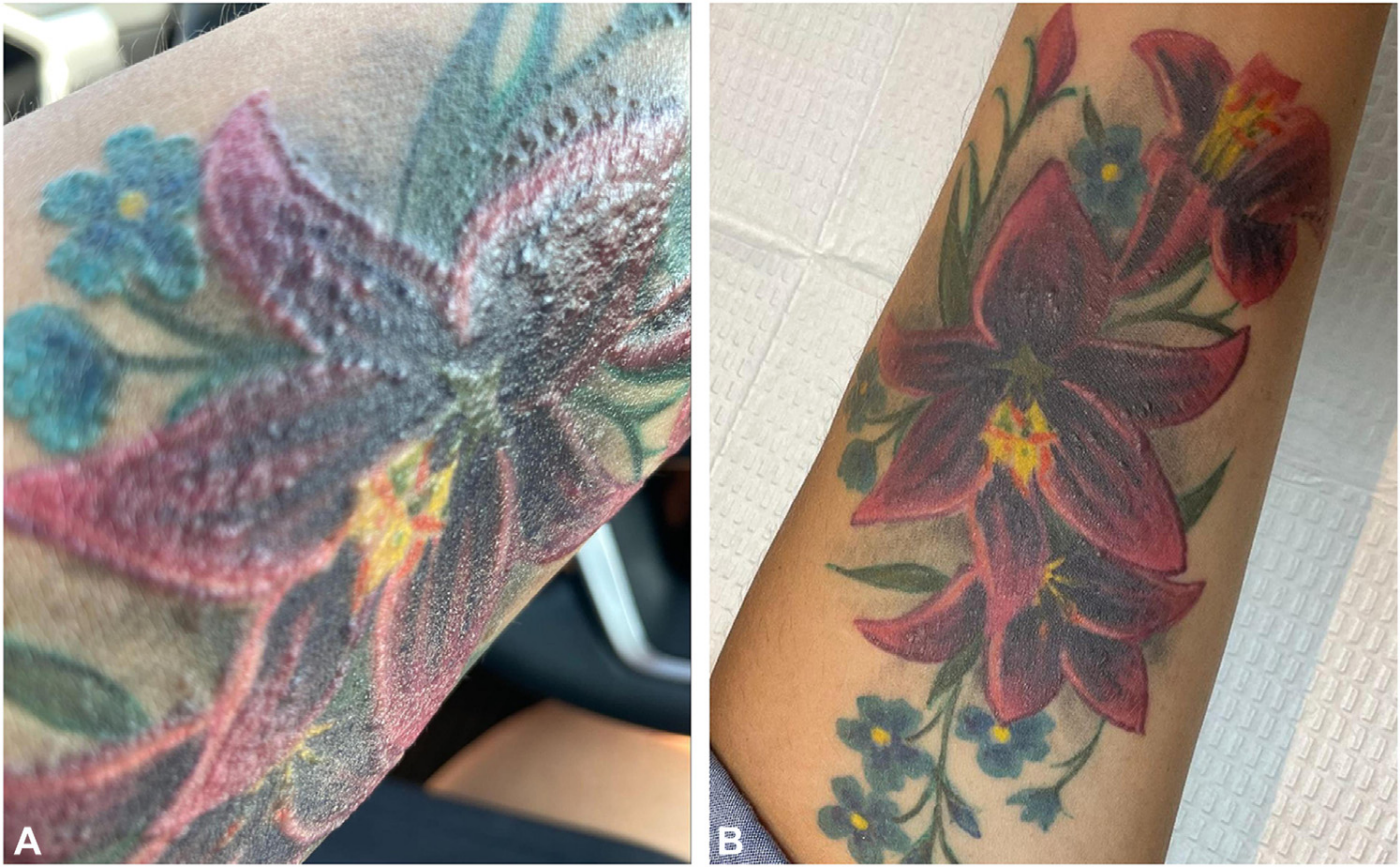
**A,** Case 2 initial presentation demonstrating erythematous nodules and eczematous plaques, (**B**) presentation at follow-up demonstrating fewer nodules.

A 47-year-old female presented to dermatology for the evaluation of a severely pruritic and raised plaque in a red tattoo located on the left proximal forearm present for 1 week. She had obtained the tattoo 7 weeks prior. The patient also reported that an orange tattoo on her back she obtained 2 years prior also became pruritic and raised at the same time as her red tattoo. The patient denied any prior problems with her tattoos and reported no significant dermatologic and medical history. The patient had used clobetasol 0.05% ointment wraps prescribed by another provider to treat the tattoo on her arm and reported improvement in her symptoms, but she was not able to discontinue treatment as the severe pruritus would recur immediately. A physical exam revealed erythematous nodules and eczematous plaques distributed on the red portion of the tattoo on her left proximal forearm (Fig 2, *A*). The patient was prescribed ruxolitinib 1.5% cream to treat the symptoms of both tattoos. At follow-up at 14 weeks, the nodules were smaller and the patient reported the ruxolitinib resolved her pruritic symptoms immediately (Fig 2, *B*). The patient reported that the clobetasol wrap alleviated pruritus and reduced the size of the raised nodules after a few days, while the ruxolitinib resolved all pruritus immediately and slightly reduced the size of the raised nodules. The patient also received intralesional triamcinolone injections to reduce the size of the raised nodules. The patient continued to use topical ruxolitinib to both tattoos to prevent new pruritus flares.

## Discussion

Tattooing is a popular global phenomenon that can occasionally be accompanied by adverse skin reactions. Notably, red tattoo ink can elicit complications presenting as allergic reactions with chronic pruritic symptoms.^1^ In a study examining the clinical aspects of red tattoo reactions, a staggering 94% of patients reported pruritic symptoms, and 17% experienced pain.^1^ Additionally, research conducted in the United States revealed an overall rate of adverse reactions from tattoos at 10.3%, with a substantial 44% of color-specific reactions attributed to red tattoo ink.^2^

Tattoo reactions, particularly those accompanied by pruritus and changes in skin texture, have a profound impact on patients' quality of life. Tattoo allergies and local reactions can present clinically with plaque-like, excessive hyperkeratotic, ulcero-necrotic, neuro-sensory, and scar patterns.^3^ Notably, the pruritus associated with these reactions is severely distressing to many patients and akin to the pruritic symptoms associated with conditions such as eczema and psoriasis.^4^^,^^5^

There are many treatments for tattoo complications documented in the literature, depending on the specific clinical presentation and severity. These interventions include removing the offending tattoo via dermatome shaving or ablative laser, or medical management of the symptoms through the use of topical and intralesional corticosteroids, and sometimes systemic therapies such as methotrexate or hydroxychloroquine.^6^, ^7^, ^8^, ^9^ Recently, a case report discussed the use of dupilumab to treat a refractory granulomatous tattoo reaction in a red tattoo.^10^

While these traditional treatment options have yielded valuable results, innovative approaches are continuously sought to address tattoo reactions. Local and intralesional corticosteroids for allergic tattoo reactions can result in skin atrophy and hypopigmentation in patients with darker skin tones.^6^ Ablative lasers can be expensive and painful, and also lead to scarring. Dermatome shaving, while effective, can also lead to scarring and there are only a limited number of dermatologists who offer it. Alternative treatment options are warranted to mitigate these risks.

To our knowledge, this case series represents the first successful instance of a tattoo reaction being effectively treated with a topical Janus kinase (JAK) inhibitor, ruxolitinib. Topical JAK inhibitors have been primarily explored for their use in conditions like atopic dermatitis, cutaneous sarcoidosis, granuloma annulare, and other granulomatous and eczematous conditions. In terms of the mechanism, ruxolitinib is a selective inhibitor of JAK1 and JAK2 which regulate the signaling in the itch response pathway through interleukin 4, interleukin 31, and thymic stromal lymphopoietin. Although the plaques and papular nodules did not completely resolve in either case, the use of ruxolitinib reduced the severity of scale and papules and effectively resolved all pruritic symptoms. The successful outcome in our 2 patients suggests that topical JAK inhibitors, such as ruxolitinib, could be a promising therapeutic option for managing tattoo-related pruritus. Further research and clinical experience are necessary to establish the safety and efficacy of JAK inhibitors in this specific context.

## Conflicts of interest

None disclosed.
